# Antimicrobial active herbal compounds against *Acinetobacter baumannii* and other pathogens

**DOI:** 10.3389/fmicb.2015.00618

**Published:** 2015-06-18

**Authors:** Vishvanath Tiwari, Ranita Roy, Monalisa Tiwari

**Affiliations:** Department of Biochemistry, Central University of RajasthanAjmer, India

**Keywords:** *Acinetobacter baumannii*, carbapenem resistance, antibacterial activity, herbal medicine, MIC

## Abstract

Bacterial pathogens cause a number of lethal diseases. Opportunistic bacterial pathogens grouped into ESKAPE pathogens that are linked to the high degree of morbidity, mortality and increased costs as described by Infectious Disease Society of America. *Acinetobacter baumannii* is one of the ESKAPE pathogens which cause respiratory infection, pneumonia and urinary tract infections. The prevalence of this pathogen increases gradually in the clinical setup where it can grow on artificial surfaces, utilize ethanol as a carbon source and resists desiccation. Carbapenems, a β-lactam, are the most commonly prescribed drugs against *A. baumannii.* The high level of acquired and intrinsic carbapenem resistance mechanisms acquired by these bacteria makes their eradication difficult. The pharmaceutical industry has no solution to this problem. Hence, it is an urgent requirement to find a suitable alternative to carbapenem, a commonly prescribed drug for *Acinetobacter* infection. In order to do this, here we have made an effort to review the active compounds of plants that have potent antibacterial activity against many bacteria including carbapenem resistant strain of *A. baumannii*. We have also briefly highlighted the separation and identification methods used for these active compounds. This review will help researchers involved in the screening of herbal active compounds that might act as a replacement for carbapenem.

## Introduction

*Acinetobacter baumannii* is a Gram-negative, non-motile, and non-fermentative coccus. It is known to cause nosocomial infections such as urinary tract infections, surgical site infection, meningitis, ventilator-associated pneumonia, bacteraemia, and very rarely intra-abdominal infections and infections of skin and central nervous system (Zarrilli et al., 2009). Clinical report showed that, the hospitalized patients who are infected with *A. baumannii* have about 30% chance of mortality (Perez et al., 2007). *Acinetobacter* are usually found in the hospital environment and infect patients who are hospitalized from a long period of time with severe underlying diseases, are immunosuppressed, or subjected to invasive procedures and treated with broad-spectrum antibiotics (Perez et al., 2007). These bacteria have been reported to colonize increasingly in the respiratory tract of patients admitted in Intensive Care Unit. In case of non-intensive care unit patients high rate of colonization of *Acinetobacter* strains are found on skin. During outbreaks along with one or more epidemic *Acinetobacter* clones, there exists an endemic strain which makes it difficult to identify and control their transmission (Marchaim et al., 2007; Oteo et al., 2007). *A. baumannii* have the ability to grow in diverse pH and temperature conditions and utilizes many different kinds of substrates for its growth (Tiwari and Tiwari, 2014). These are known to survive in both dry and moist condition and on inanimate objects like contaminated medical instruments including ventilators, catheters, respirometers (Cunha et al., 1980; Cefai et al., 1990; Horrevorts et al., 1995), pillows (Weernink et al., 1995), bed mattresses (Sherertz and Sullivan, 1985) etc. They are found widely in water and soil and some of them are also isolated from animals (Manchanda et al., 2010). Numerous cases of worldwide outbreaks due to *A. baumannii* depict increasing rates of resistance of these bacteria toward commercially available antibacterial agents.

Multi-drug resistant *A. baumannii* have been reported to be isolated from the hospitals in India (Tiwari et al., 2012; Tiwari and Moganty, 2013, 2014; Tiwari and Tiwari, 2015), Turkey (Vila et al., 2007), Taiwan (Lin et al., 2011), Argentina (Merkier and Centron, 2006), Korea (Chaulagain et al., 2012), Japan (Endo et al., 2012), Iran (Shahcheraghi et al., 2011), Saudi Arabia (Alsultan et al., 2009), Latin America, North America, Europe, Asia-Pacific rim (Mendes et al., 2010; Wang and Dowzicky, 2010), Brazil (Dalla-Costa et al., 2003; Gales et al., 2003; Sader et al., 2005; Tognim et al., 2006; Villegas et al., 2007), many countries of Asia and Middle-East (Afzal-Shah et al., 2001; Abbo et al., 2005; Jeon et al., 2005; Jeong et al., 2006; Chan et al., 2007; Ko et al., 2007; Koh et al., 2007), and also from Australia and Pacific islands (Anstey et al., 1992, 2002; Riley et al., 1996; Peleg et al., 2006; Iredell et al., 2007; Playford et al., 2007; Valenzuela et al., 2007). Previous reports suggested that some MDR strains of *A. baumannii* spread from regions of Spain (Culebras et al., 2010), with high antibacterial resistance rates, to regions like Norway, experiencing low rates of resistance. It was also reported that the US military personnel and civilians have received medical support in United Kingdom when they were infected by the MDR strains, while posted in an operation in Iraq and Afghanistan (Perez et al., 2007). Gradually since the late 1970s, cases of resistance started developing against most classes of drugs. In the late 1990s, the only effective therapeutic option left was carbapenem. In recent years, *A. baumannii* was also found to become resistant to carbapenem making its treatment more and more difficult. Various resistance mechanisms have been acquired by *A. baumannii* against carbapenem (Manchanda et al., 2010), such as presence of antimicrobial-inactivating enzymes, i.e., β-lactamase (Brown et al., 2005; Chuang et al., 2010; Rodriguez-Martinez et al., 2010; Acosta et al., 2011; Santella et al., 2011), decreased access to bacterial targets (because of reduced permeability of the outer membrane caused by loss of or low porin expression, increased expression of multidrug efflux pumps) (Fernandez-Cuenca et al., 2003; Wilke et al., 2005; Hu et al., 2007; Vila et al., 2007; Vashist et al., 2010), and mutations altering targets or different cellular functions. These mechanisms may act in combination also for a single strain (Fernandez-Cuenca et al., 2003; Rice, 2006).

Although no antibiotics with specific cellular targets have been isolated from plants but modified plant natural antibiotics has been more successful such as penicillin (Lewis and Ausubel, 2006). Attempts to develop potent antibiotics from plants have failed by both pharmaceutical and biotech firms. A reason for this may be the use of varying chemical strategy by the plants to control bacterial infection, in order to reduce the selective pressure for developing resistance to antibiotics. For instance, antibacterial active compounds may act quite effectively in combinations and have little potency alone. Plant alkaloid berberine has excellent antibacterial properties but it is ineffective when acting alone as it is a preferred substrate of bacterial encoded MDRs (multidrug resistance pumps). 5′-methoxyhydnocarpin, a compound isolated from the same plant as berberine blocks the MDRs and therefore enabling berberine to act as a potent antibacterial agent in presence of this compound. So it is very necessary to first identify the antibacterial mechanism of the plants and then screen pharmaceutically to develop some effective antibiotic (Lewis and Ausubel, 2006).

Since there is no new development of antibiotics against the carbapenem resistant strains of *A. baumannii* therefore, it is necessary to focus on the antimicrobial activity of plant derived substances that are being used in traditional medicine worldwide (Savoia, 2012). Secondary metabolites are responsible for the antimicrobial activity of plants. In this present review, we have explained the various herbal active compounds which have potent activity against *A. baumannii* and other Gram-negative bacteria. This will initiate the search for new antibiotics from herbal origin against the resistant strains of *Acinetobacter baumannii* and this may lead to the development of new antibiotics.

## Extraction of plant active compounds and its identification

Numerous works have been done to isolate and characterize bioactive compounds from plant resources that are active against Gram-positive and Gram-negative bacteria, fungi and viruses. Miyasaki reported that generally flavones, tannins and phenolic compounds are known to be active against *Acinetobacter* (Miyasaki et al., 2013). Many active herbal compounds have been isolated and reported for their activities. These secondary metabolites were extracted using different solvents such as methanol, ethanol, water, hydroxide, acetone, and through various techniques like HPLC, column chromatography from plant materials. After extraction of the plant materials, elucidation of the active metabolites and their structures were performed by different methods. The methods include GC-MS (gas chromatography-mass spectrometer), LC-MS (liquid chromatography-mass spectrometer), and NMR (nuclear magnetic resonance).

The efficiency for separating and identifying compounds from complex biological mixture is very high for GC-MS (Villas-Boas et al., 2005). It is capable of simultaneously identifying and profiling several compounds that possess different functions. This is mostly used for making the fingerprints of compounds that are volatile in nature which somewhat make the analyzing process repetitive and time consuming (Formisano et al., 2012; Huang et al., 2012; Wang et al., 2012). GC-MS can be used for compounds having lower molecular mass and that are volatile or semi volatile in nature (Villas-Boas et al., 2005; Dunn et al., 2011). Here, the capillary column has a low flow rate, so that output of the column can be directly put to MS ionization chamber (Oleszek and Marston, 2000; Phillipson, 2007; Daffre et al., 2008). Besides GC-MS, another most important analytical technique is LC-MS where liquid chromatography is coupled to mass spectrometry along with electrospray ionization and atmospheric pressure chemical ionization (Villas-Boas et al., 2005). It can identify and analyze a large number of compounds, present in small quantities whose molecular mass vary from 10 to 300,000 with varying chemical properties (Hagel and Facchini, 2008). As compared to GC-MS, sample preparation is quite simple in case of LC/MS (Dunn and Ellis, 2005). By LC/MS analysis, high resolution mass accuracy can be achieved which is important for analyzing herbal active compounds. This property is enhanced by the use of ultra-performance liquid chromatography. It can also be said that LC-MS is more preferred over GC-MS in reducing time and money for sample preparation. NMR spectra provide great information about the structures of the compounds along with their identification via interpretation of spectra involving chemical shift and chemical constants. It is quantitative, reproducible, non-sample destructive, and non-specific which makes it superior to other analytical techniques (Dunn et al., 2011).

The antibacterial components of various plants, their separation and identification techniques are listed below in Table 1. Some of these herbal compounds have the ability to work alone or in presence of the other, i.e., in synergy which are also explained in Table 1.

**Table 1:** **Antibacterial components of plants with their extraction and identification methods**.

**Plant (Common name)**	**Antibacterial compounds**	**Extracted from**	**Separation methods**	**Identification methods**	**References**
*Rosa rugosa* (Japanese rose)	Ellagic acid	Dry powders of plant extract	HPLC	LC/MS, NMR	Miyasaki et al., 2013
*Terminalia chebula* (Myrobalan/Hardad)	Chebulagic acid, Chebulinic acid, Corilagin, and Terchebulin	Dry powders of plant extract	HPLC	LC/MS, NMR	Miyasaki et al., 2013
*Scutellaria baicalensis* (Baical skullcap)	Norwogonin, Baicalin, and Baicalein	Dry powders of plant extract	HPLC, Aqueous and ethanolic extract	LC/MS, NMR	Chan et al., 2011; Miyasaki et al., 2013
*Lythrum salicaria* (Purple loosestrife)	Hexahydroxy diphenoyl ester vescalagin	Flowers and leaves	Methanolic extract, Column chromatography	N/A	Becker et al., 2005; Guclu et al., 2014
*Syzygium aromaticum*^a^^,^^b^ (Clove)	Eugenol	Essential leaf oil	Hydroxide solution extraction and distillation	N/A	Pelletier, 2012; Saulnier, 2014
*Cinnamomum zeylanicum*^a^^,^^b^ (Cinnamon/Dalchini)	Trans-cinnamaldehyde	Essential leaf oil	N/A	N/A	Pelletier, 2012; Saulnier, 2014
*Oreganum vulgare*^a^^,^^b^ (Oregano)	Carvacrol	Essential leaf oil	N/A	N/A	Pelletier, 2012; Saulnier, 2014
*Thymus*^b^ (Common thyme/Garden thyme)	Thymol	Essential leaf oil	N/A	N/A	Pelletier, 2012
*Curcuma longa*^c^ (Turmeric/Haldi)	Curcumin	Plant extract powder	Methanolic extract	N/A	Betts and Wareham, 2014
*Camellia sinensis*^c^^,^^d^ (Green tea)	Epigallocatechingallate, epicatechin	Plant extract powder	Ethanolic extract	N/A	Betts et al., 2011; Betts and Wareham, 2014
*Camellia sinensis*^d^ (Black tea)	Theaflavin	Plant extract powder	Ethanolic extract	N/A	Betts et al., 2011
*Lycium chinense* Mill. (Cortex lycii/Wolfberry)	(+)-Lyoniresinol-3 alpha-O-beta-D-glucopyranoside	Herbal materials	Aqueous and ethanolic extract	N/A	Chan et al., 2011
*Paeonia suffruticosa* Andr. (Cortex moutan)	Paeonol	Herbal materials	Aqueous and ethanolic extract	N/A	Chan et al., 2011
*Coptidis chinensis* Franch. (Rhizoma coptidis)	Berberine	Herbal materials	Aqueous and ethanolic extract	N/A	Chan et al., 2011
*Berberis fremontii* (Desert barberry)	Berberine	Leaves	Hexane extract	NMR, MS	Stermitz et al., 2000; Lewis and Ausubel, 2006
*Hydrastis Canadensis* (Goldenseal)	Berberine	Aerial parts	Aqueous and ethanolic extract	LC-MS	Lewis and Ausubel, 2006; Ettefagh et al., 2011
*Azadirachta indica* (Neem)	stigmasterol, nimbiol, sugiol, 4-cymene, α-terpinene, terpinen-4-ol	Leaves, bark	Methanolic extract	GC-MS	Nand et al., 2012
*Aloe vera* (Indian aloe, Ghi Kunvar)	p-coumaric acid, ascorbic acid, pyrocatechol cinnamic acid	Leaves	ethanol, methanol and acetone extracts, thin layer and column chromatography	GC-MS	Lawrence et al., 2009
*Allium sativum* (Garlic)	allyl methyl disulfide, diallylsulfide, diallyltrisulfide, allyl methyl trisulfide, diallyl disulfide	Bulbs	HPLC	GC-MS	Khadri et al., 2010; Lu et al., 2011
*Magnolia dealbata* (Cloudforest magnolia)	Honokiol, magnolol	Seeds	Ethanolic extract	N/A	Jacobo-Salcedo Mdel et al., 2011
*Rabdosia rubescens* (Blushred rabdosia)	phaeophytin a, phaeophytin b, 17c-ethoxypheaophorbide a, 17c-ethoxyphea ophorbide b, oleanolic acid, physcion, emodin-8-O-beta-D-glucopyranoside, isorhamnetin	N/A	ODS column chromatography	NMR and MS	Lv and Xu, 2008

a Syzygium aromaticum, Cinnamomum zeylanicum, Oreganum vulgare shows synergism.

b Syzygium aromaticum, Cinnamomum zeylanicum, Oreganum vulgare and Thymus shows synergism.

c Curcuma longa and Camellia sinensis (Green tea) exhibits synergism.

d Camellia sinensis (Green tea) and Camellia sinensis (Black tea) exhibits synergism.

## Plant active compounds against gram-negative bacteria

The antibacterial components of various plants were screened against a wide range of Gram-positive as well as Gram-negative bacteria mostly by MIC, disk diffusion assay and CFU. It was found that some of the compounds were more active against Gram-positive whereas some were more active against Gram-negative bacteria. The Gram-positive bacteria that are tested against different active compounds of plants include *Staphylococcus aureus*, *Bacillus cereus*, *Helicobacter pylori, Salmonella enteritidis*, and *Clostridium jejuni* (see Table 2). The Gram-negative bacteria like *Klebsiella pneumoniae*, *Enterobacter aerogenes*, *Providencia stuartii*, *Escherichia coli*, *Enterobacter cloacae*, and *Pseudomonas aeruginosa* are susceptible toward the active compounds of *Hibiscus subdarifa, L. salicaria, Adansonia digitata, Commiphora molmol*, etc. (see Table 2).

**Table 2:** **List of bacteria susceptible to the bioactive herbal components**.

**Active compounds**	**Source**	**Antibacterial assay method**	**Active against bacteria**	**MIC (μg/ml)**	**References**
Hexahydroxy diphenoyl ester vescalagin	*Lythrum salicaria*	Agar well diffusion test	*A. baumannii*	N/A	Becker et al., 2005; Guclu et al., 2014
			*H. pylori*	N/A	
			*P. aeruginosa*	N/A	
			*Staphylococcus aureus*	62	
			*B. cereus*	N/A	
			*Mycobacterium smegmatis*	N/A	
			*Micrococcus luteus*	125	
			*Proteus mirabilis*	62	
Ellagic acid	*Rosa rugosa*	MIC, MBC	*A. baumannii*	250	Miyasaki et al., 2013
Terchebulin	*Terminalia chebula*	MIC, MBC	*A. baumannii*	500	Miyasaki et al., 2013
Chebulagic acid				1000	
Chebulinic acid				62.5	
Corilagin				1000	
Norwogonin	*Scutellaria baicalensis*	MIC, MBC	*A. baumannii*	128	Chan et al., 2011; Miyasaki et al., 2013
Baicalin				N/A	
Baicalein				N/A	
Eugenol	*Syzygium aromaticum*	MIC, CFU	*A. baumannii*	1250	Kollanoor Johny et al., 2010; Pelletier, 2012; Saulnier, 2014
			*C. jejuni*	N/A	
			*S. enteritidis*	N/A	
Trans-cinnamaldehyde	*Cinnamomum zeylanicum*	MIC, CFU	*A. baumannii*	310	Kollanoor Johny et al., 2010; Pelletier, 2012; Saulnier, 2014
		CFU	*S. enteritidis*	N/A	
		CFU	*C. jejuni*	N/A	
Carvacrol	*Oreganum vulgare*	MIC, CFU	Biofilms of *Staphylococcus epidermidis*	0.031%, v/v	Nostro et al., 2007; Kollanoor Johny et al., 2010; Pelletier, 2012; Saulnier, 2014
			Biofilms of *S. aureus*	0.015–0.031%, v/v	
			*A. baumannii*	310	
			*S. enteritidis*	N/A	
			*C. jejuni*	N/A	
Thymol	*Thymus*	CFU, MIC	*S.* enteritidis	N/A	Nostro et al., 2007; Kollanoor Johny et al., 2010; Pelletier, 2012
			*C. jejuni*	N/A	
			*A. baumannii*	N/A	
			*S. epidermidis* biofilms	0.031%, v/v	
			biofilms of *S. aureus*	0.031–0.062%, v/v	
Curcumin	*Curcuma longa*	MIC, time kill assay, FIC index, CFU	*S. aureus*	125–250	De et al., 2009; Hu et al., 2013; Mun et al., 2013; Betts and Wareham, 2014
			*H. pylori*	5–50	
			*A. baumannii*	4 (in presence of EGCG)	
			*Streptococcus mutans*	175	
Epigallocatechin gallate (EGCG)	*Camellia sinensis* (Green tea)	MIC, time kill assay, FIC index, CFU	*S. aureus*	100	Kono et al., 1997; Zhao et al., 2002; Osterburg et al., 2009; Gordon and Wareham, 2010; Betts and Wareham, 2014
			*A. baumannii*	312–625	
			*Stenotrophomonas maltophilia*	512	
			*H. pylori*	64	
Epicatechin	*Camellia sinensis* (Green tea)	Disk diffusion assay	*A. baumannii*	N/A	Betts et al., 2011
			*S. maltophilia*	N/A	
Theaflavin	*Camellia sinensis* (Black tea)	Disk diffusion assay	*A. baumannii*	N/A	Betts et al., 2011
			*S. maltophilia*	N/A	
(+)-Lyoniresinol-3 alpha-O-beta-D-glucopyranoside	*Lycium chinense* Mill.	CFU	*A. baumannii*	N/A	Chan et al., 2011
			*S. aureus*	N/A	
			*Enterococcus faecalis*	N/A	
Paeonol	*Paeonia suffruticosa* Andr.	CFU	*A. baumannii*	N/A	Chan et al., 2011
			*S. aureus*	N/A	
			*E. faecalis*	N/A	
Berberine	*Coptidis chinensis* Franch.	CFU	*A. baumannii*	N/A	Chan et al., 2011
			*S. aureus*	N/A	
			*E. faecalis*	N/A	
Berberine	*Berberis fremontii* (Desert barberry)	MIC	*S. aureus*	30	Stermitz et al., 2000; Lewis and Ausubel, 2006
Berberine	*Hydrastis Canadensis* (Golden seal)	CFU, FIC	*S. aureus, Streptococcus pyogenes*	N/A	Lewis and Ausubel, 2006; Ettefagh et al., 2011
Honokiol, Magnolol	*Magnolia dealbata*	Disk diffusion assay	*Clavibacter michiganensis, P. aeruginosa, A. baumannii, Acinetobacter iwoffii*	N/A	Jacobo-Salcedo Mdel et al., 2011
α-elemene, δ-elemene, furanosesquiterpenes	*Commiphora molmol* (Myrrh)	Disk diffusion assay, CFU, MIC	*K. pneumoniae*	6250	Masoud and Gouda, 2012
			*P. aeruginosa*	6250	
			*A. baumannii*	2500	
			*E. coli*	6250	
p-Coumaric acid, ascorbic acid, pyrocatechol, cinnamic acid	*Aloe vera*	Agar well Diffusion Technique	*S. aureus, Streptococcus pyogenes, B. subtilis, Bacillus cereus, E. coli, P. aeruginosa, K. pneumonia, Salmonella typhi*	N/A	Lawrence et al., 2009
Allyl methyl disulfide, Diallylsulfide, Diallyltrisulfide, Allyl methyl trisulfide, Diallyldisulfide, Diallyltetrasulfide	*Allium sativum* (Garlic)	MIC	*P. aeruginosa*	3120	Khadri et al., 2010; Lu et al., 2011
			*K. pneumonia*	1250	
			*A. baumannii*	3120	
		CFU	*C. jejuni*	N/A	
Stigmasterol, Nimbiol, sugiol,4-cymene, α-terpinene, terpinen-4-ol	*Azadirachta indica*	Disk diffusion assay	*S. epidermidis*	N/A	Fabry et al., 1998; Nand et al., 2012
		MIC	*S. aureus*	1000	
			*Enterococci*	500	
			*Klebsiella*	2000	
			*P*. *aeruginosa*	1000	
			*E. coli*	2000	
			*Salmonella*	2000	
Gossypetin, hibiscetin, quercetin, sabdaretin, delphinidin 3-O-sambubioside and cyanidin 3-O-sambubioside	*Hibiscus subdarifa* (Indian sorrel/Rose mallow)	MIC, MBC	*K. pneumoniae*	1024	Djeussi et al., 2013; Pacôme et al., 2014
			*E. aerogenes*	1024	
			*P. stuartii*	512	
			*E. coli*	1024	
			*E. cloacae*	256	
Quercetin-7-0-B-D-xylopyranoside, 7-baueren-3-acetate	*Adansonia digitata* (Baobab/Gorakh imli)	MIC, MBC	*K. pneumoniae*	1024	Djeussi et al., 2013
			*E. coli*	1024	
			*E. cloacae*	1024	
			*Providencia stuartii*	1024	
			*E. aerogenes*	1024	

MIC, Minimum Inhibitory Concentration; MBC, Minimum Bactericidal Concentration; FIC, Fractional Inhibitory Concentration; CFU, Colony Forming Units.

## Plant active compounds against *Acinetobacter baumannii*

This study is aimed to review the literature regarding herbal active compounds against various bacteria, including *Acinetobacter baumannii*. Hence, we have listed most of the plants in Table 2, which are reported to inhibit this pathogen by varying mechanisms. *L. salicaria* shows significant activity against different bacteria but, especially *A. baumannii* and *P. aeruginosa.* Hence its topical form may be used to treat infections of skin and soft tissue (antiseptic), infections of burn wounds, diabetic foot and decubitus wound caused by these MDR bacterial strains. It is already known that this plant has been used as traditional medicine for many indications, but to use it clinically, several *in vitro* and *in vivo* test have to be performed(Guclu et al., 2014).

Saulnier et al. used essential oils of herbs like *Syzygium aromaticum, Cinnamomum zeylanicum*, and *Thymus* in nano medicine against multidrug-resistant *A. baumannii*. Cinnamaldehyde prevents the activity of amino acid decarboxylase in the bacteria (Burt, 2004), but it is unable to disorganize outer membrane of cell or deplete intracellular ATP concentration. Hydroxyl group of carvacrol and eugenol (phenolic compounds) can disrupt the bacterial cell wall. This phenomenon has the potential to decrease intracellular ATP pool and membrane potential (Ultee et al., 2002; Gill and Holley, 2006). It also results in the leakage of various substances such as ATP, amino acids, ions, and nucleic acids ultimately leading to bacterial death. MIC of active components is the same as MIC of those compounds when nano-encapsulated. Lipidic nanocapsules (LNC) can be made more effective by improving their presence in systemic circulation through the modifications on LNC surface. It can be a good alternative of antibiotics (Saulnier, 2014).

Curcumin alone had very little antibacterial activity against *A. baumannii* strains with high MIC (256 μg/ml). The antibacterial activity of curcumin is due to several reasons for instance, disruption of folic acid metabolism (shikimate dehydrogenase) pathway (De et al., 2009) and bacterial cell division (Rai et al., 2008). Combinatorial use of curcumin and epigallocatechin gallate (EGCG) is very much effective in increasing the inhibition level by many folds, making the MIC 4 μg/ml. This study indicates synergistic effects to prevent *A. baumannii* growth between curcumin and EGCG without any antagonistic effects (Betts and Wareham, 2014). In the same way epicatechin, a tea polyphenol having no antibacterial properties can potentiate theaflavin, increasing its activity against *A. baumannii* and *S. maltophilia* isolates. The probable mechanism may be that epicatechin inhibits theaflavin oxidation thus enhancing its antibacterial effect, but the exact mechanism of synergy is not yet understood and needs further study (Betts et al., 2011).

Khadri, et al. reported the presence of methyl allyl trisulfide (34.61%) and diallyl disulfide (31.65%) with other compounds at relatively lower levels after GC/MS analysis in *Allium sativium.* It showed significant activity against *P. aeruginosa in vitro* and can be used for treating infections caused by this pathogen (Khadri et al., 2010). *Aloe vera* gel extract were reported to be more active for Gram-positive than Gram-negative bacteria. Ethanol extract was most active followed by methanol extract activity and least inhibition was exhibited by acetone extract (Lawrence et al., 2009). *Magnolia dealbata* extracts showed good inhibition zone of >10 mm against *P. aeruginosa, Clavibacter michiganensis, A. baumannii, A. iwoffii.* Generally, the active constituents of *M. dealbata* like honokiol and magnolol possess selective antimicrobial activities against drug resistant Gram-negative bacterial species and fungal pathogens (Jacobo-Salcedo Mdel et al., 2011).

Miyasaki et al. (2013) suggested in their studies, norwogonin (5,6,7-trihydroxyflavone) extracted from *Scutellaria baicalensis*, has an MIC_90_ of 128 μg/ml against some strains of *A. baumannii*. Chebulagic acid, chebulinic acid (65% inhibition at 62.5 μg/ml), ellagic acid (67% inhibition at 250 μg/ml), corilagin, and terchebulin extracted from *Terminalia chebula* had lower activity against *A. baumannii in vitro*. Other constituents of *Scutellaria baicalensis*, baicalin and baicalein are also found to be active against other bacteria. Corilagin, chebulagic acid, and terchebulin of *Terminalia chebula* exhibits a two-step killing kinetic. The medical literature reported that many phenolic compounds of plant extracts enhance the potential of synthetic antibiotics against *A. baumannii in vitro* (Miyasaki et al., 2013). For instance, activity of rifampicin, coumermycin, fusidic acid, novobiocin, and chlorobiocin was enhanced by tannic acid and ellagic acid against *A. baumannii in vitro* (Chusri et al., 2009). Even synergy was observed between topical mafenide and green tea polyphenol against multi-drug resistance *Acinetobacter baumannii in-vitro* (Osterburg et al., 2009), while no effect of synergy was noted between any antibiotics for Gram-negative bacteria and norwogonin.

Some Chinese medicines extracted from different plants have been reported to exhibit significant antibacterial activities. The active constituent of *Rhizoma coptidis* is berberine, an alkaloid possessing various antimicrobial activities. Berberine is also isolated from *Berberis fremontii* and *Hydrastis canadensis* (Lewis and Ausubel, 2006) It has anti-Herpes simplex virus effects and at moderate concentrations (30–45 μg/ml) sufficient antibacterial effect was observed along with inhibition of biofilm formation. Plant derived antibacterial molecule are generally weak but work better in synergy with antibiotics (Lewis and Ausubel, 2006). Synergism was also seen for berberine and β-lactam antibiotics against multi drug resistant *S. aureus*. (+)-Lyoniresinol-3 alpha-*O*-beta-D-glucopyranoside of Cortex Lycii presented strong antimicrobial effect against multi drug resistant *S. aureus* isolated from patients and some pathogenic fungi, but it did not cause any haemolysis on human RBCs. This also possesses potent antifungal activities against *Candida albicans.* There is very limited literature concerned with the antibacterial effects of Cortex Moutan. The ethanolic extract of Cortex Moutan suppress the growth of *S. aureus* (Chan et al., 2011). Only paeonol is identified as active ingredients till date, which is responsible for its anti-microbial effects on *C. albicans, C. tropicalis*, and *C. glabrata* etc. (Chan et al., 2011).

A very common medicinal plant *A. indica* is the source of various active compounds identified by GC/MS analysis possessing versatile effects like anti-bacterial, anti-inflammatory, antioxidant activities (Nand et al., 2012). Hence further studies can be performed to see its activity against Gram-negative bacteria and especially *A. baumannii.*

## Conclusion and future prospects

Emergence of resistant strains of bacterial pathogens is a major source of high morbidity, mortality, and increased cost, making its treatment much more difficult. Traditionally, plants play an important role in treatment of diseases, hence it may be used as a source to find out alternative drug to carbapenem. Plants possess secondary metabolites that are reported to be potentially active against a wide variety of bacteria. It was also found that the herbal compounds are weak antibacterials but showed better antimicrobial activity when used in synergy with other antibiotics. The advancement in the techniques for separation, purification and identification of bioactive compounds make it possible to chemically and structurally identify these compounds. In present review, we have made an effort to list a number of plant active compounds that may have potent antibacterial activity against some Gram-positive and Gram-negative bacteria including carbapenem resistant strain of *A. baumannii*. Hence, the use of different plant natural compounds as antibacterial agents is an interesting strategy for discovering bioactive products that could become useful therapeutic tools. This review will help researchers involved in the screening of herbal active compounds that might act as a replacement for carbapenem, a β-lactam. In this review we found a compound named norwogonin which has good antibacterial activity (MIC_90_ – 128 μg/ml) against *A. baumannii.* Hence, special emphasis can be given on this compound in further studies in order to use it as an alternative of carbapenem. Effect of this compound on other bacteria can also be screened. There are some plants shown in Table 2 that shows good activity against many bacteria like *Allium sativum*, *Adansonia digitata*, *Hibiscus subdarifa*, etc., have not been tested against *A. baumannii.* Therefore, activities of these plants can also be tested against *A. baumannii*. Screened herbal active compound may be tested in the future for its antimicrobial activity. *In-silico* approach can also be used to modify the structure of active compound for its better antibacterial activity against carbapenem resistant strain of *A. baumannii*. Potential plant active compound may be chemically synthesized and used as an alternative to carbapenem.

### Conflict of interest statement

The authors declare that the research was conducted in the absence of any commercial or financial relationships that could be construed as a potential conflict of interest.
